# Crowberry inhibits cell proliferation and migration through a molecular mechanism that includes inhibition of *DEK* and Akt signaling in cholangiocarcinoma

**DOI:** 10.1186/s13020-022-00623-6

**Published:** 2022-06-13

**Authors:** Xue Wang, Xuebing Zhou, Ludan Zhang, Xin Zhang, Chunyu Yang, Yingshi Piao, Jinhua Zhao, Lili Jin, Guihua Jin, Renbo An, Xiangshan Ren

**Affiliations:** 1 Department of Pathology and Cancer Research Center, Yanbian University, Jilin Yanbian, 133002 China; 2 Key Laboratory of Pathobiology, Yanbian University, State Ethnic Affairs Commission, Yanji, China; 3 Key Laboratory of Natural Medicines of the Changbai Mountain (Yanbian University), Ministry of Education, Jilin Yanbian, 133002 China; 4 Department of Immunology and Pathogenic Biology, Yanbian University, Yanji, 133002 China

**Keywords:** Cholangiocarcinoma, *Empetrum nigrum* var. *japonicum*, Proliferation, Migration, *DEK*

## Abstract

**Background:**

Cholangiocarcinoma (CCA) is a rare biliary adenocarcinoma related to poor clinical prognosis. Crowberry is an herbal medicine used to control inflammatory diseases and reestablish antioxidant enzyme activity. Although crowberry shows significant therapeutic efficacy in various tumors and diseases, its anticancer effects and specific molecular mechanisms in CCA are poorly understood.

**Aim of the study:**

This study was conducted to characterize crowberry effects on CCA cells behavior.

**Materials and methods:**

The chemical profiles of crowberry extract was qualitatively analyzed by high-performance liquid chromatography (HPLC) and HPLC–tandem mass spectrometry. MTT, colony formation and EdU assays were performed to measure cell proliferation. The effect of crowberry treatment on CCA cell migration was assessed by wound healing and migration assays. Moreover, Hoechst staining assay and flow cytometry were performed to assess the cell apoptosis rate. Western blotting was used to assess the protein expression levels of key factors associated with apoptosis, the Akt signaling pathway, and the epithelial-mesenchymal transition. A xenograft model was established and immunohistochemical and H&E staining was performed to assess crowberry antitumor effects in vivo.

**Results:**

Crowberry clearly inhibited CCA cells proliferation and migration in a dose-dependent manner and induced apoptosis in vitro. Crowberry inactivated the PI3K/Akt signaling pathway by regulating *DEK *in vitro and significantly inhibited tumor growth by downregulating the *DEK* expression in xenograft models*.*

**Conclusion:**

Crowberry inhibits CCA cells proliferation and migration through a molecular mechanism that includes inhibition of *DEK* and Akt signaling pathway inhibition in vitro and in vivo.

**Graphical Abstract:**

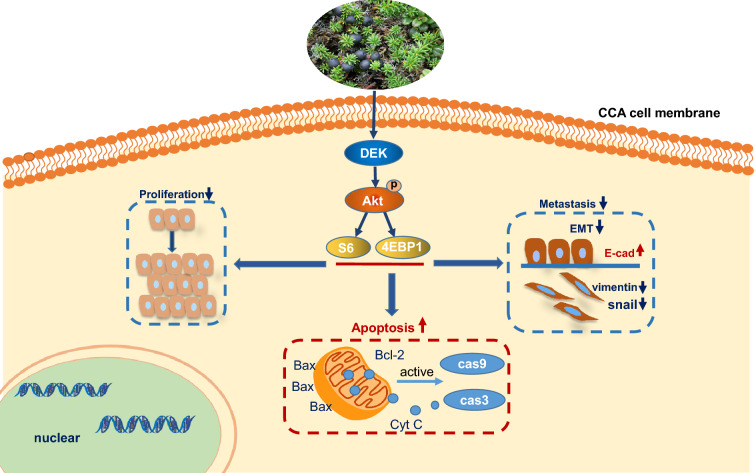

**Supplementary Information:**

The online version contains supplementary material available at 10.1186/s13020-022-00623-6.

## Highlights

1. Crowberry alterd expression levels of key mediators in PI3K/Akt signaling pathway.
2. Crowberry alterd expression levels of key mediators in PI3K/Akt signaling pathway.
3. Crowberry suppressed the expression of the proto-oncogene *DEK *in vivo and in vitro.
4. Crowberry inhibited CCA progression and migration through a molecular mechanism that includes inhibition of *DEK* and the Akt signaling pathway in vivo and in vitro.

**Supplementary Information:**

The online version contains supplementary material available at 10.1186/s13020-022-00623-6.

## Introduction

Cholangiocarcinoma (CCA) is a malignant cancer that originates from the bile duct epithelial cells and is associated with poor outcomes. Even after major clinical interventions, such as surgery, radiation therapy, and chemotherapy, the survival rate is not significantly improved and remains low with only 15% surviving to five years after treatment [1, 2]. Because of poor clinical outcomes, more effective therapeutic options that induce low toxicity are urgently required.

*Empetrum nigrum* var. *japonicum* Siebold & Zucc. ex K.Koch, also known as crowberry, is a small class of creeping evergreen shrubs and an undomesticated bacca that has considerable herbal potential [3]. It exhibits both anti-inflammatory and anticancer functions. For example, it has been used to maintain youth and treat inflammatory diseases, such as cystitis and urethritis [4, 5]. Moreover, ultraviolet B radiation-induced cell damage can be repaired by crowberry extract treatment [6]. In addition, recent studies have shown that crowberry contains important bioactive compounds [7, 8], and several of these components, including (2S)-5'-hydroxy-7,3',4'-trimethoxyflavanone, 2',4'-dihydroxy-3'-methoxychalcone, 4'-hydroxy-7-methoxyflavanone and 2',4'-dihydroxychalcone, have shown strong anticancer effects on P-388 lymphoid leukemia and HT-29 colon cancer cell [9]. In addition, crowberry is recorded in *Hulunbeier Mongolian Medicine Resources*, because its branches and leaves can be used as supplements to treat the spleen and stomach, particularly treat indigestion, and the fruit nourishes the liver; People often use crowberry as a medicine for the prevention and treatment of alcoholic liver disease, and it reportedly has a satisfactory effect as a Chinese folk [10]. Although significant therapeutic effects of crowberry on various diseases and tumors have been reported, the specific molecular mechanisms of crowberry action in CCA are not well understood.

*DEK* is an oncoprotein on human chromosome 6p22.3 of. Acetylation reduces the affinity of *DEK* for DNA and inhibits deacetylase resulting in the accumulation of particle clusters *DEK* (IGC) in chromatin and RNA processing factors comprising subnuclear structures. Subsequently, through the abovementioned complex posttranslational modifications, the following processes are realized: RNA transcription, RNA splicing, DNA replication and DNA repair [11]. *DEK* has been proven to promote the formation and development of solid tumors: for instance, *DEK* is highly expressed in breast cancer and is associated with a malignant phenotype and progression [12]. High *DEK* expression can promote the epithelial-to-mesenchymal transition (EMT) process of esophageal squamous carcinoma cells, accelerate lymph node metastasis, and significantly reduce the overall survival rate of patients [13]. High expression of *DEK* in pancreatic cancer has been found to promote lymph node metastasis and to be related to poor prognosis [14]. Additionally, the overall survival rate of liver cancer patients with high *DEK* expression is significantly reduced [15]. However, *DEK* expression is rarely reported in CCA. The Akt/mTOR pathway is essential for the proliferative and migratory potential of cells. Various diseases, such as inflammation and cancer, can develop when signaling through the Akt/mTOR pathway is inefficient [16, 17]. Natural plants that can attenuate the liver fibrosis by regulating the phosphorylation of components in the Akt/mTOR pathway have attracted attention [18]. Actually, the GeneMANIA database indicates that there is a significant connection between *DEK* and the Akt/mTOR signaling pathway. *DEK* participates in tumor cell proliferation, apoptosis invasion and differentiation by regulating several signaling pathways, such as Akt signaling pathways, thereby promoting tumor cell growth and infiltration [19, 20]. These studies indicate that *DEK* can potentially be used as a target for the development of antitumor drugs.

The main purpose of this study was to characterize the inhibitory effect of crowberry on the HuCCT1 and QBC939 CCA cell lines and elucidate the molecular mechanism of its action. Taken together, this study provides new ideas and a theoretical basis for the clinical treatment of CCA.

## Materials and methods

### Plant materials and extraction

Crowberry was provided by the Key Laboratory of Natural Medicines of the Changbai Mountain (Yanbian University), Ministry of Education. The whole plant of crowberry was purchased in March 2018 at the Gen He Crude drug market in Hei Long Jiang Province, China. The voucher specimen (No. YBU-1020) was identified by Professor Hui-zi Lv, Botanical garden, Yanbian University, and stored in the Herbarium of the College of Pharmacy at Yanbian University (China). The air-dried and powdered whole plant of crowberry (6.0 kg) were extracted with MeOH (25 L, reflux, 5 h × 3), followed by removal of the solvent under reduced pressure to yield a dried MeOH extract (1.5 kg, yield 25%). The dried MeOH extract was dissolved in dimethyl sulfoxide (DMSO) to a density of 300 mg/ml and deposited at − 20 °C until analysis.

### High-performance liquid chromatography and HPLC–tandem mass spectrometry (MS/MS)

**Fig. 1:**
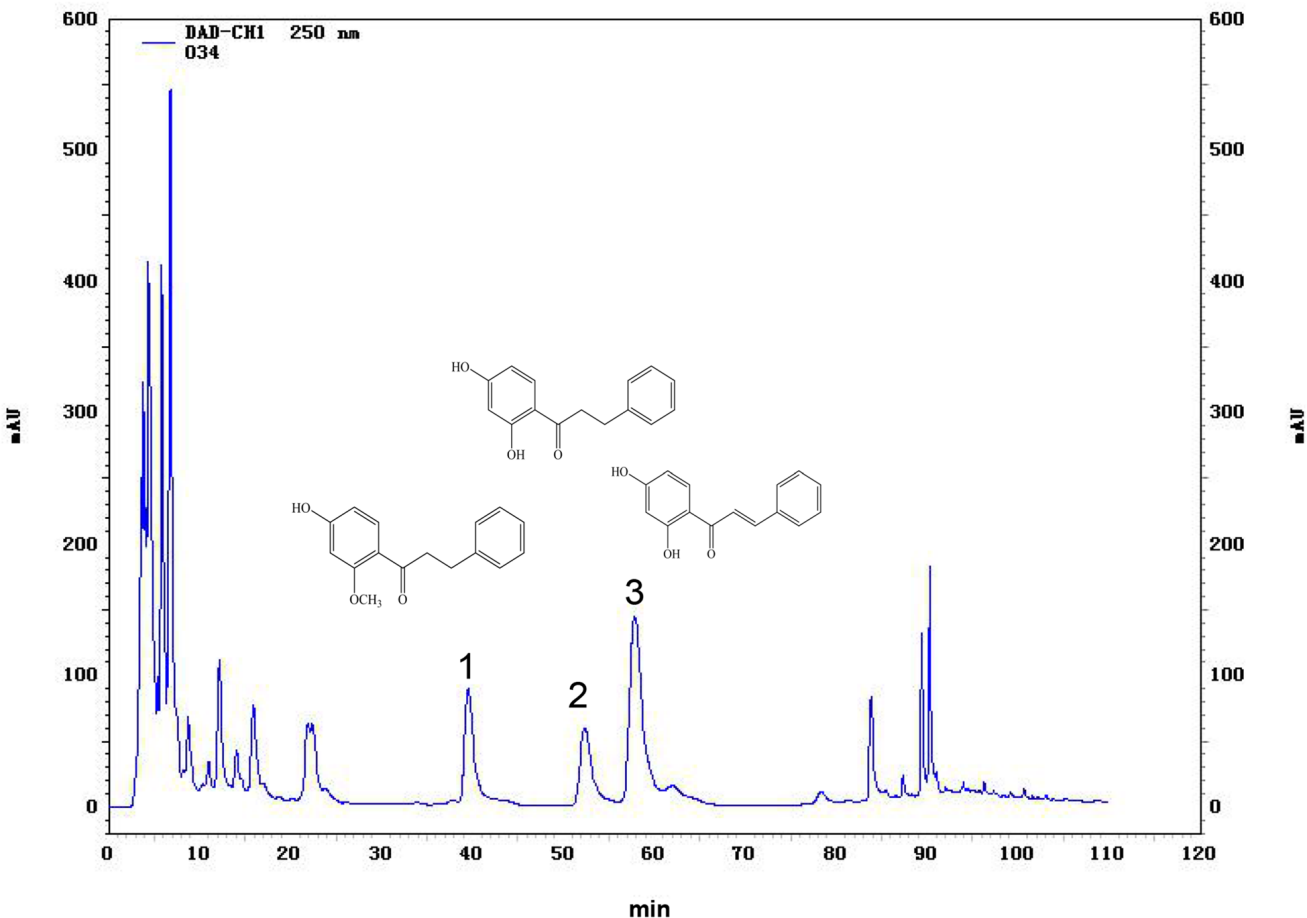
Bioactive compounds in crowberry extract. Structures of three flavonoid monomers and HPLC profiles of crowberry extract sample solution. 2′-methoxy-4′-hydroxy-dihydrochalcone (**1**), 2′,4′-dihydroxy-dihydrochalcone (**2**), and 2′,4′-dihydroxy-chalcone (**3**) were determined at 250 nm

Chromatographic experiments were performed using an HPLC instrument equipped with a 5260 autosampler and 5430 diode-assay UV/Vis detector (DAD) (HITACHI Chromaster, Japan) and mass spectrometry (Agilent, 6460, USA). For all experiments, a LaChrom ODS C-18 column (250 mmL × 4.6 mmI.D., 5 μm, HITACHI, Japan) was used as the stationary phase, and the syringe amount was 5 μL. The mobile phase was composed of 0.5% phosphoric acid solution (A) and methanol (D), and the system was equilibrated for 20 min with the beginning conditions. The following gradient program has been applied to methanol (D): linear gradient from 58% D to 60% D for 20 min, 60% D increasing to 62% D in 55 min, and then 62% D increasing to 100% D for 20 min. The column was washed with 100% D for 10 min, and the flow rate was 0.7 mL/min. UV absorption at 250 nm was measured to define the analyses (Fig. 1). Then obtain the corresponding chromatographic peaks for 2'-methoxy-4'-hydroxy-dihydrochalcone(1), 2',4'-dihydroxy-dihydrochalcone(2), and 2',4'-dihydroxy-chalcone (3).

### Cell lines and cell culture

CCA cell lines, RBE and QBC939, were purchased from the Cell Bank of Chinese Academy of Sciences (Shanghai, China). The human cholangiocarcinoma cell lines, HuCCT1 and TFK1, were gifted by Kanazawa University in Japan. Normal human bile duct epithelial cell HIBEpic was obtained from American Type Culture Collection (ATCC, Manassas, VA, USA). Cell lines above were grown in RPMI-1640 medium (GIBCO, Gaithersburg, MD). Cells were supplemented 1% penicillin/streptomycin and 10% fetal bovine serum (GIBCO, Gaithersburg, MD). Cells were incubated at 37 °C with a 5% CO2 atmosphere.

### MTT assay

HuCCT1 and QBC939 cells were seeded 5000 cells/well in a 96-well plate, and treated with crowberry at various concentrations for 24, 48, 72 h. Correspondingly, silenced and overexpressed *DEK* cells were cultured for 48 h. Briefly, the value of absorbance was measured at a full-wavelength spectrophtotmeter (TECAN-infinite M200 pro, Switzerland).

### Colony formation assay

Cells were seeded in six-well plates and then cultured with crowberry for 2 weeks. The visible colonies were fixed with 4% paraformaldehyde and stained with 1% crystal violet (Solarbio, China). ImageJ software was used to analysze the data.

### EdU assay

Cells were seeded at 5000 cells/well in 96-well plates. After treatment, these cells were cultured in complete medium containing 50 µM EdU (RiboBio, China) for 2 h at 37 °C followed by washing twice with phosphate-buffered saline (PBS). The cells were fixed and permeabilized and then, EdU was detected following the EdU assay kit manufacturer’s instructions. Finally, cell immunostaining was observed and images were acquired with a fluorescence microscope (Olympus, Japan).

### Hoechst33342 staining

After treating with the indicated crowberry for 48 h, cells were fixed with 4% formaldehydefor, and stained for 15 min with 1 μg/ml Hoechst33342 in the dark. The cells were then washed twice with PBS. A fluorescence microscope was used to image the cells.

### Flow cytometry

Cells treated with different concentrations of crowberry after 48 h. The cells were washed with PBS and re-suspended in binding buffer. Cells were stained in the dark with FITC-annexin V and PI (BD Biosciences, USA) for 15 min. BD Accuri C6 (BD Biosciences) was used to detect the samples.

### Wound healing assay

Cells were cultured in 6-well plate to 80% confluence. The wounds were created by 200 µl micropipette tip and replaced with different doses of crowberry. Cell migration was observed at 0, 12 and 48 h using microscopy (Olympus, Japan). Silenced or overexpressed *DEK* cells were observed at 12 h. Image J software was used to analyze the results.

### Cell migration assay

Cells were seeded in the upper chambers of Transwell inserts (Costar, Corning Incorporated, USA), at a concentration of 5 × 10^4^ cells/well. Then the cells were incubated with medium containing 1% fetal bovine serum (FBS) for 4 h. Medium containing 10% or 20% FBS was added to the lower chamber. Specifically, QBC939 cells were cultured for 72 h, and HuCCT1 cells were cultured for 24 h. Cells were fixed with 4% paraformaldehyde and then stained with 1% crystal violet. Cells were photographed with an Olympus BX53 microscope.

### Transfection

*DEK* small interfering RNA (siRNA) reagent was purchased from RiboBio (China). The effective sequence of the *DEK* gene used here was (5'-TGTCCTCATTAAAGAAGAA-3). The cells were incubated with 50 nM siRNAs containing Lipofectamine 3000 (Invitrogen, Carlsbad, CA) for 48 h. An lentiviral vector overexpressing *DEK* (*DEK* (Human, NM_003472)) was purchased from Beijing Syngentech Co., Ltd, China, which serial number is pHS-AVC-0466. HuCCT1 cells were transfected with this vectorin a 6-well plate, and 2 ml of medium supplemented with 2 µl of polybrene and 2 µl of lentivirus was added for transfection. After 8 h on incubation, the medium was replaced with fresh medium, and the cells were incubated for another 72 h. The *DEK*-transduced cells were incubated with 5 μg/ml puromycin in the medium.

### Western blotting

After the cells were treated with crowberry for 48 h, total proteins (40 μg) were quantified with BCA protein assay (Beyotime, Shanghai, China), electrophoresed through SDS-PAGE, and transferred to PVDF membrane (Millipore, Eschbom, Germany). The membrane was incubated with primary antibodies Bax (1:1000), Bcl-2 (1:1000), Cleaved-Caspase3 (1:1000), Cleaved-Caspase9 (1:1000), Cleaved-PARP (1:1000), Cytoc (1:1000), p-Akt (1:1000), Akt (1:1000), p-S6 (1:1000), S6 (1:1000), p-4EBP1 (1:1000), 4EBP1 (1:1000), E-cadherin (1:1000), Vimentin (1:1000), and Snail (1:1000), which were obtained from Cell Signaling Technology, USA, and *DEK* (1:1000, BD, USA), actin (1:3000, Abcam, USA) at 4 ℃ overnight. Secondary antibody (1:3000, Beyotime, China) was added at RT for 1 h. Enzymatic signals were visualized with a ChemiDoc Touch Imaging System (Bio-Rad, USA), and statistics were performed with ImageJ software.

### Xenograft model

The study was approved by the Ethics Committee of Yanbian University. BALB/C female nude mice (4 weeks old) were obtained from Changzhou Cavens Laboratory Animal Co., Ltd., China. Each mouse was injected with 2 × 10^6^/100 µl QBC939 cells subcutaneously into the rigtht armpit. One week after inoculation, 16 mice were randomly assigned into 2 groups (8 mice in each group). Every day, mouse body weight was recorded, and tumors were measured with a Vernier caliper; the tumor volume was ascertained following the formula: tumor volume = 0.5 × (length × width^2^). The treatment group was treated every day with crowberry (50 mg/kg) diluted with 0.1% methylcellulose for intragastric administration (ig), and the same volume of methylcellulose was used to treat the control group mice. On day 30, the mice were anesthetized with 1% pentobarbital sodium and then sacrificed. Mouse livers and tumors were fixed in 4% paraformaldehyde for immunohistochemical and hematoxylin and eosin (H&E). This experiment was completed in a specific-pathogen-free (SPF) animal laboratory in the Yanbian University Animal Experiment Center. The experiment was approved by the Animal Protection Committee of Yanbian University (SCXK (JI)2017–0003). the research was conducted in accordance with internationally accepted principles (European community guidelines) for laboratory animal use and care.

### Immunohistochemistry staining (IHC)

The fixed mouse tumors were embedded in paraffin and then sliced into 4 µm thin slices. Antigen was retrieved in 100 °C citrate buffer for 10 min. After endogenous peroxidase was blocked with 1% hydrogen peroxide for 10 min, the Ki67 antibody (1:100, Affinity Biosciences, rabbit monoclonal, USA) and DEK antibody (1:100, protheintech, Mouse monoclona, USA) were incubated at 4 ℃ overnight. After incubation with secondary antibody, the section was observed with diaminobenzidine (DAB, Zhongshan Golden Bridge PV- 9000, Beijing, China), and the nucleus was stained with hematoxylin. Five fields of 400 × view were selected to count the number of Ki67-positive cells.

### Statistical analysis

Statistical analyses were primarily conducted by GraphPad Prism 8.0 software (GraphPad, San Diego, CA) and SPSS 20.0 software (SPSS, Chicago, IL). One-way ANOVA was used for analysis multiple comparisons. Two group comparisons were performed using T-test. *P-Values* < 0.05 were considered as significant.

## Results

### Bioactive compounds in crowberry extract

The existence of flavonoid monomers of the chemical compounds in the methanol extract of crowberry extract has been shown using HPLC–MS/MS analysis. Figure 1 shows the HPLC chromatograms recorded at 250 nm, the main dominant compound as 2',4'-dihydroxy-chalcone. The contents of 2'-methoxy-4'-hydroxy-dihydrochalcone (**1**), 2',4'-dihydroxy-dihydrochalcone (**2**), and 2',4'-dihydroxy-chalcone (**3**) were 0.71%, 1.15% and 1.35%, respectively.

### Crowberry suppresses the proliferation and colony formation of CCA cell lines

**Fig.2:**
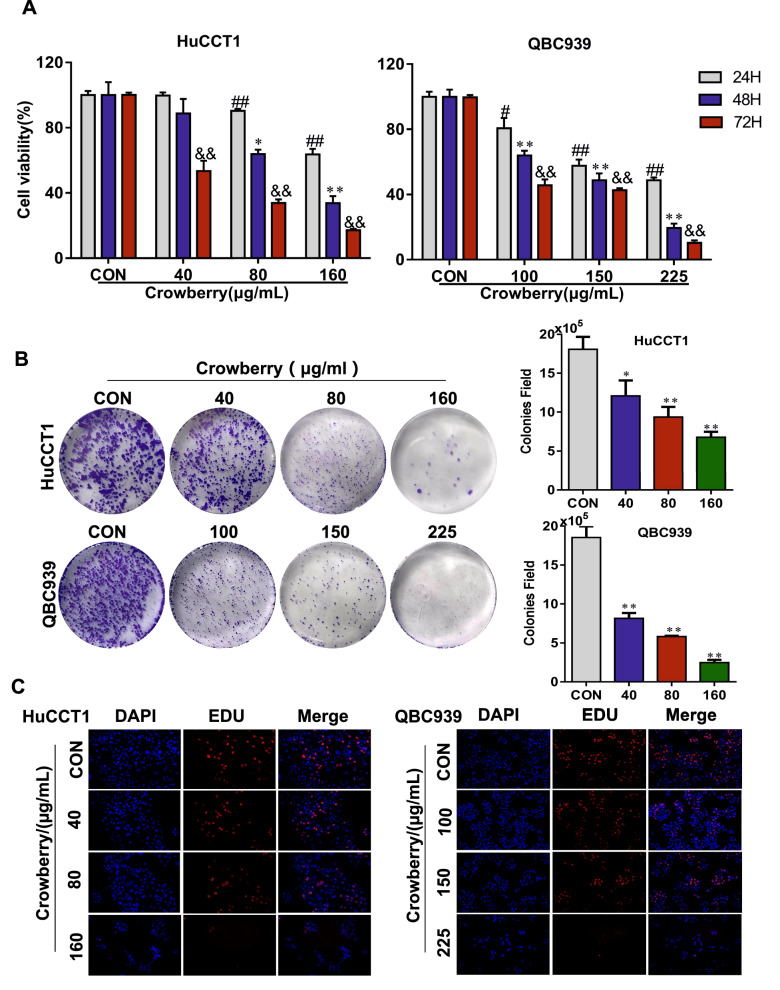
Crowberry suppresses the proliferation and colony formation of CCA cell lines. MTT assay was used to detect the effect of crowberry on the viability in HuCCT1 and QBC939 cells (**A**). Colony formation assay to detect the ability of CCA cells (**B**). EdU assay to detect the proliferation of CCA cells after treatment with crowberry (**C**). *P < 0.05, **P < 0.01 (vs CON group, n = 3); CON, control

Polyphenolic compounds are a major group of secondary metabolites that have the anticancer property. To assess the inhibitory effects of crowberry on CCA cells, the 3-(4,5-dimethylthiazol-2-yl)-2,5 diphenyltetrazolium bromide (MTT) assay was used to detect cell viability. HuCCT1 and QBC939 cells were treated with different concentrations of crowberry. The result showed that crowberry profoundly inhibited the cell viability of HuCCT1 and QBC939 cells in a dose- and time-dependent manner compared with control group cells. Then the half-inhibition rate (IC50) of HuCCT1 (87.5 µg/ml) and QBC939 (144.1 µg/ml) were calculated. Accordingly, the concentrations of crowberry selected for further experiments were 40, 80, and 160 µg/ml for HuCCT1 and 100, 150, and 225 µg/ml for QBC939 cells (*P* < 0.05 or* P* < 0.01; Fig. 2A). Then, the proliferative ability was determined using colony formation and 5-ethynyl-2'-deoxyuridine (EdU) assays, and the results showed that crowberry significantly reduced the proliferation of HuCCT1 and QBC939 cells in a dose-dependent manner compared with control group cells (*P* < 0.01; Fig. 2B, C). In conclusion, crowberry inhibits the proliferation of CCA cells.

### Crowberry induces the apoptosis of CCA cells

**Fig.3:**
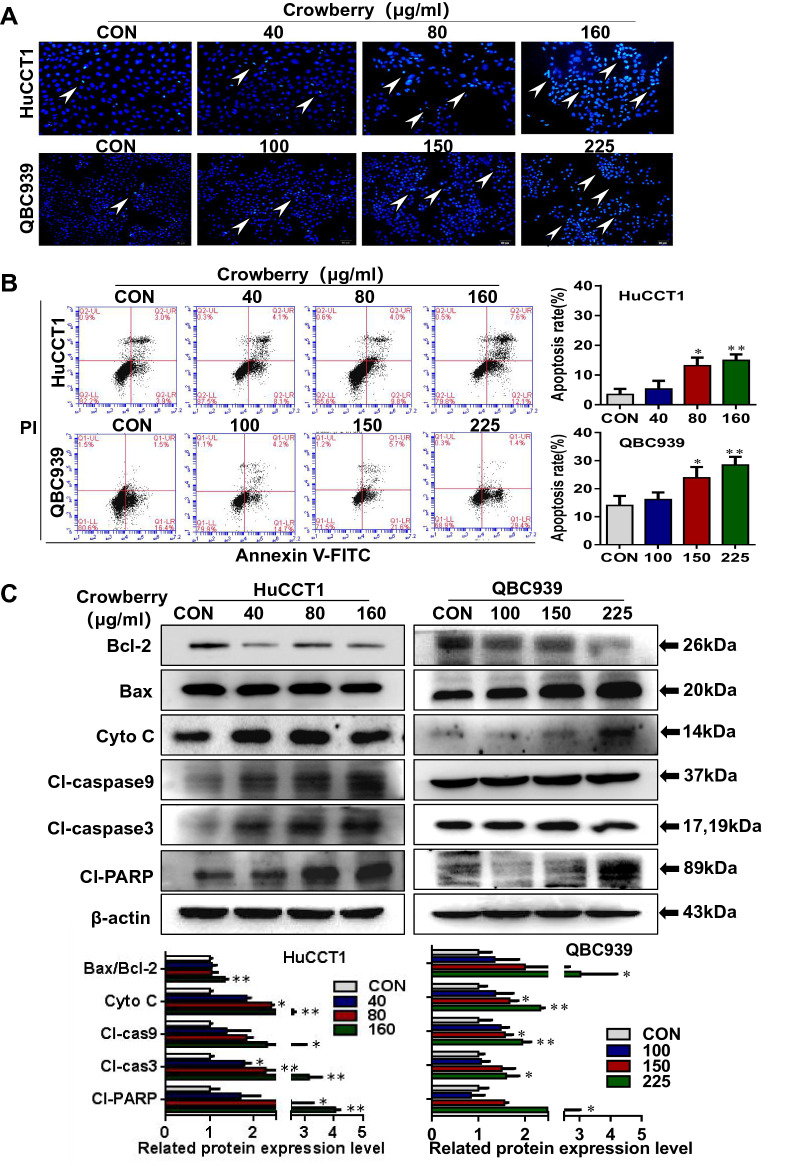
Crowberry induces the apoptosis of CCA cells. Hoechst33342 staining (white arrow) (**A**) and flow cytometry (**B**) were performed to detect the apoptosis of CCA cells. The expression of apoptosis marker proteins in CCA cells and statistical chart of western blotting assays (**C**). *P < 0.05, **P < 0.01 (vs CON group, n = 3). Original magnifications, × 200 (**A**). CON, control

To determine whether crowberry has an apoptotic effect on CCA cell lines, we performed Hoechst 33,342 staining, which revealed an obvious induction of apoptosis in HuCCT1 and QBC939 cells following crowberry treatment (Fig. 3A). Similarly, the results of Annexin-V and PI staining showed that the number of apoptotic cells increased significantly after treatment with crowberry compared to the control cells (*P* < 0.05 or *P* < 0.01; Fig. 3B). Moreover, crowberry significantly increased the level of pro-apoptotic proteins compared with control group cells, including cleaved caspase-9, cleaved caspase-3, cleaved-PARP, and Bax. Similarly, crowberry treatment also increased the level of key mitochondrial apoptotic pathway factor Cyto C, whereas the expression of the anti-apoptotic gene *Bcl2* was clearly reduced (Fig. 3C). These consequences demonstrate that crowberry induces the apoptosis of CCA cells.

### Crowberry suppresses the migration and EMT of CCA cells

**Fig.4:**
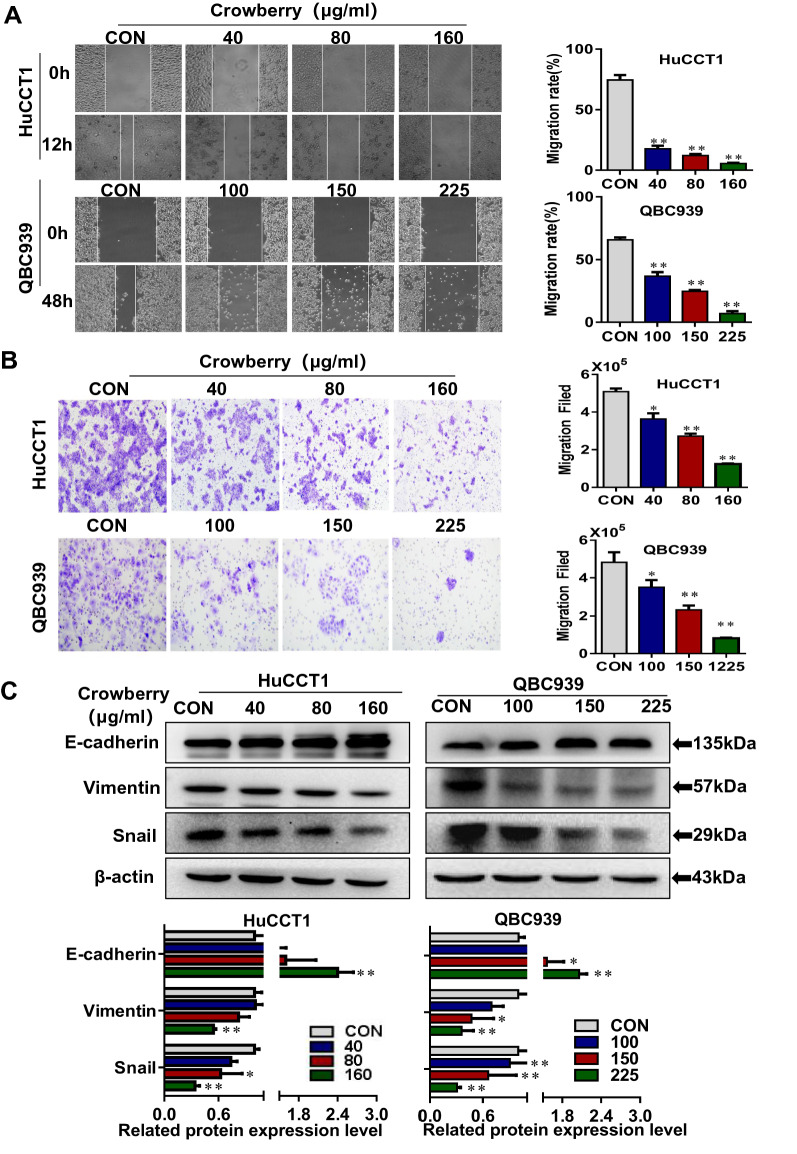
Crowberry suppresses the migration and EMT of CCA cells. Effect of crowberry on the horizontal distance of HuCCT1 and QBC939 cells (**A**). Vertical migration ability of cells (**B**). The expression of EMT marker proteins after treatment with crowberry and statistical chart of western blotting assays (**C**). *P < 0.05, **P < 0.01 (vs CON group, n = 3). × 40 (**A**), × 200 (**B**). CON, control

Given the close connection between EMT and tumor migration [21], we examined the effects of crowberry on EMT in CCA cells using wound healing, transwell, and western blotting assays. Crowberry significantly reduced the horizontal and vertical migration of CCA cells in vitro contrast to control group cells (*P* < 0.01; Fig. 4A, B), as defined by wound healing and transwell assays. Furthermore, crowberry increased the expression of E-cadherin, whereas it inhibited the level of Vimentin and Snail (Fig. 4C). To prove that the suppressed migration was not from suppression of cell growth or cell death caused by the relatively high concentration, HuCCT1 and QBC939 cells were treated with lower concentration of crowberry, followed by MTT assay and wound healing assay. The results showed that when the concentrations were 20 µg/ml and 50 µg/ml, the cell viability of HuCCT1 and QBC939 cells was not affected (*P* < 0.01; Additional file 1: Fig. SA), but the horizontal migration ability was significantly inhibited (*P* < 0.01; Additional file 1: Fig. SB). These results indicate that crowberry prevents EMT and migration in CCA cells.

### Crowberry inhibits the Akt/mTOR signaling pathway

**Fig.5:**
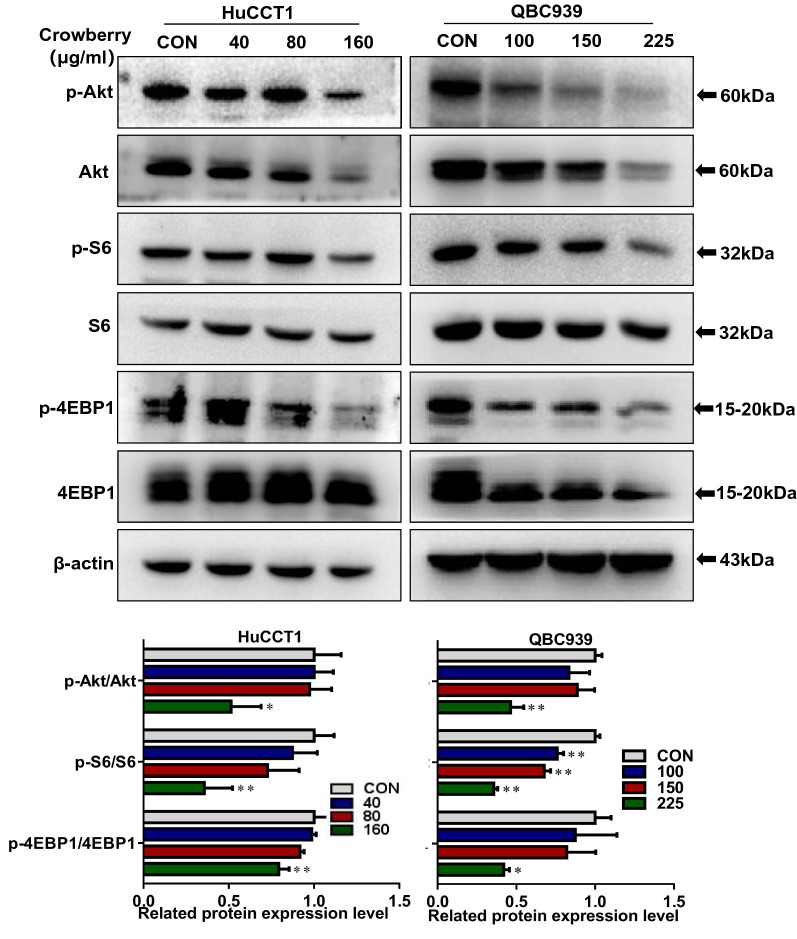
Crowberry inhibits Akt/mTOR signaling pathway. The key marker in Akt signaling pathway was tested by western blotting, after treatment with crowberry and the statistical chart of western blotting assays. *P < 0.05, **P < 0.01 (vs CON group, n = 3). CON, control

We next examined the level of key mediators of the Akt/mTOR signaling pathway. Crowberry treatment obviously decreased the phosphorylated level of Akt, S6, and 4EBP1 compared with control group cells (Fig. 5), suggesting that crowberry attenuates the proliferative potential of CCA cell by inhibiting Akt/mTOR pathway.

### Crowberry suppresses cell proliferation and migration by regulating DEK

**Fig.6:**
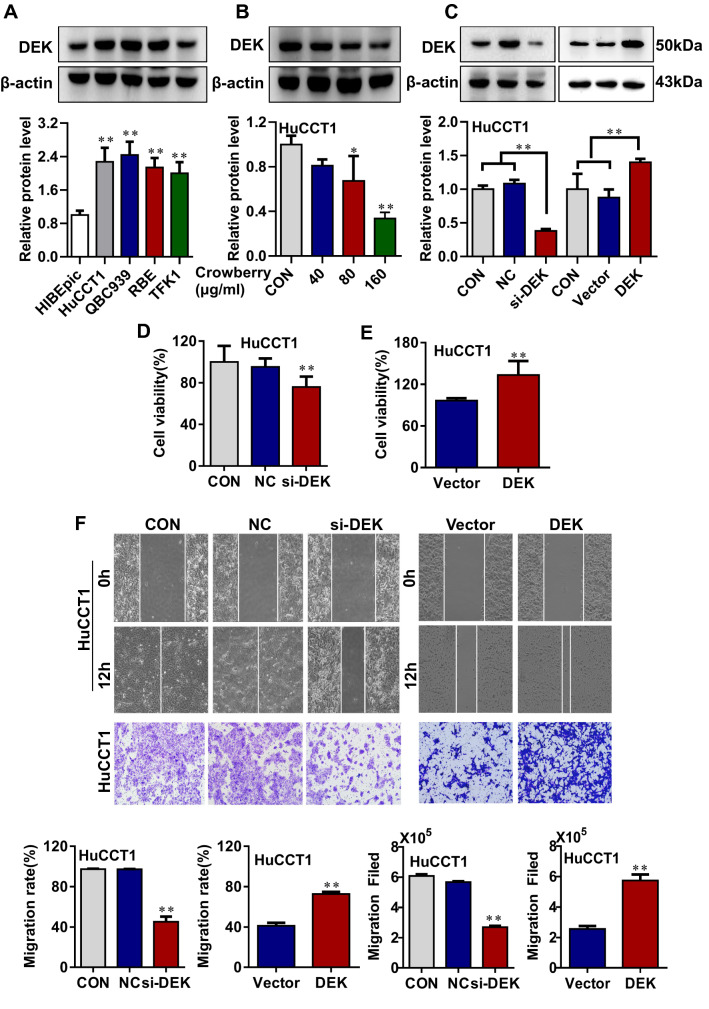
Crowberry suppresses cell proliferation and migration by regulating *DEK.* The expression of *DEK* in HIBEpic, HuCCT1, QBC939, REE, TFK1 and after treatment with crowberry for 48 h in HuCCT1 was dectected by western blotting. The statistical chart of western blotting assays is attaching (**A** and **B**). The effect of gene silencing and overprseeion was presented by western blotting (**C**). After the *DEK* gene was silenced and overpressed, the effects on cell proliferation, migration shown in (**D**–**F**). *P < 0.05, **P < 0.01 (vs CON in si*DEK* group, vs Vector in overexpression group, n = 3). Original magnifications, × 40 (**F**). Original magnifications, × 200 (**G**). CON, control. NC, negative control

*DEK* plays important roles in the proliferation, migration, and metastasis of tumor cells [22, 23]. Due to understand the molecular mechanism of crowberry action in detail, we detected *DEK* expression between HIBEpic and CCA cells. Figure 6A demonstrated that *DEK* significantly upregulates in CCA cells compared with HIBEpic cells. Additionally, the levels of *DEK* significantly reduced after being treated with crowberry (Fig. 6B). We then silenced and overexpressed *DEK* in HuCCT1 cells to demonstrate the potential oncogenic role of *DEK* in CCA cells (Fig. 6C). As a result, a significant reduction in cell proliferation was observed in si*DEK* cells and the result in overexpression *DEK* cells was reversed (Fig. 6D, E). Moreover, lateral and longitudinal migration abilities were significantly weakened compared with control group. In contrast, the migration abilities of HuCCT1 cells were significantly enhanced after overexpression of *DEK* (Fig. 6F). Above all, these data indicated that *DEK* is overexpressed in CCA cell lines, and cowberry suppression of *DEK* expression may contribute to its roles in CCA inhibition.

### Crowberry targets DEK and Akt/mTOR signaling pathway to inhibit EMT progression in CCA cells

**Fig.7:**
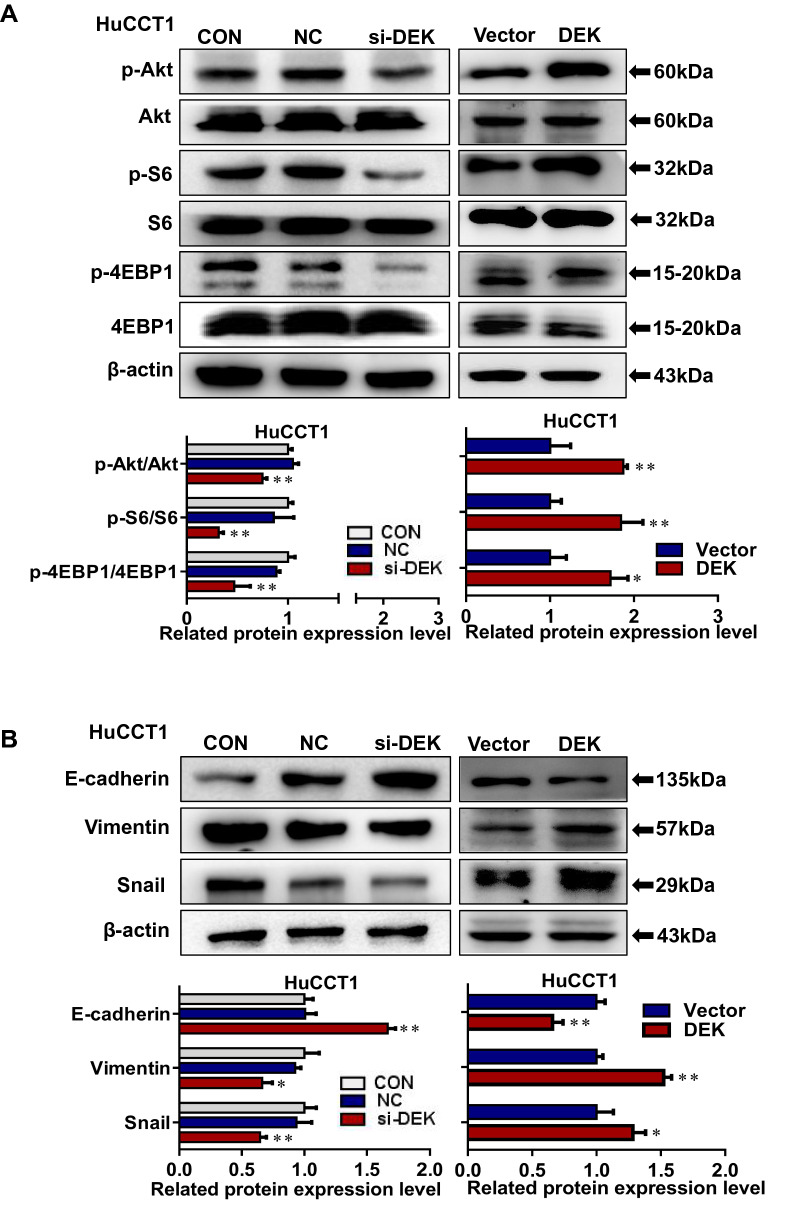
Crowberry targets *DEK* and Akt/mTOR signaling pathway to inhibit EMT progression in CCA cells. Results of p-Akt, Akt, p-S6, S6, p-4EBP1 and 4EBP1 were detected by silenced and overexpressed *DEK* in HuCCT cell (**A**); Expressions of E-cadherin, Vimentin, Snail were detected by silenced and overexpressed *DEK* in HuCCT cell (**B**). The statistical chart of western blotting assays is attaching. CON, control. NC, negative control. *P < 0.05, **P < 0.01 (vs CON in si*DEK* group, vs Vector in overpression group, n = 3)

Furthermore, *DEK* silencing downregulated p-Akt, p-4EBP1, and p-S6 expression compared with control group cells, and overexpression cells were reversed (*P* < 0.05 or *P* < 0.01; Fig. 7A). Additionally, E-cadherin expression was upregulated after *DEK* silencing, whereas the Vimentin and Snail expression were downregulated compared with control group cells. As we speculated, the results were reversed in HuCCT1 cells overexpression *DEK* (Fig. 7B). It implied that *DEK* exerts oncogenic functions via Akt signaling pathway modulationin CCA cells.

### *Crowberry inhibits CCA progression in vivo*

**Fig.8:**
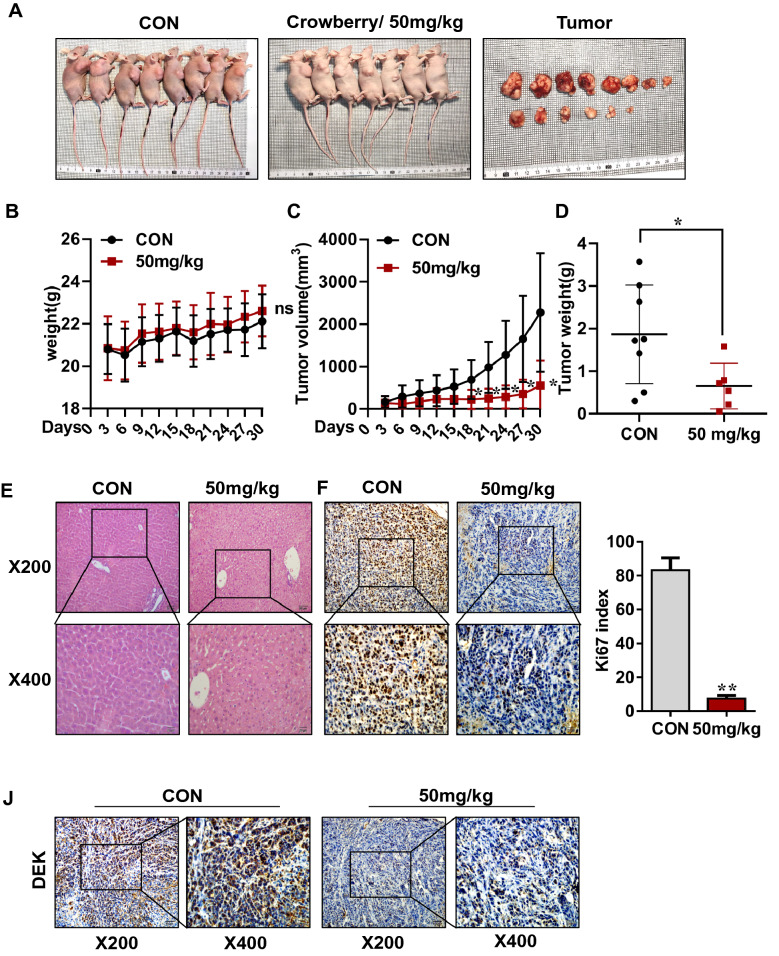
Crowberry inhibits CCA progression in vivo*.* Body and tumor-bearing representative images of mice (**A**); Changes of mouse body weight over time after crowberry treatment (**B**); Changes in tumor volume over time after crowberry treatment (**C**); The effect of using crowberry on tumor weight (**D**); H&E staining of liver to assess the toxicity of crowberry (**E**); The expression of *DEK* and Ki67 in mouse tumor tissues illustrated by IHC staining (**F**–**J**). **P < 0.01 (vs CON group, n = 5), CON, control. ns, no significance

To further convince the vitro observations, xenograft model was performed. The general view of nude mice and dissected tumors are shown in Fig. 8A. Unfortunately, one mouse of 50 mg/kg group died during ig. Another mice in treatment group has no tumor, and was not included in the statistics. Compared with the control group, there was no significant change in the weight of the nude mice (Fig. 8B). Figure 8C, D showed that the 50 mg/kg group tumors exhibited a significant reduction in volume and weight (*P* < 0.01) compared with control group. To evaluate the toxic effect of crowberry, H&E staining was performed. The results showed that the liver fiber was intact without inflammatory cell infiltration (Fig. 8E), suggesting that there was no damage of crowberry at a dose of 50 mg/kg. Further, the IHC staining was performed, indicating that Ki67 and *DEK* were significantly decreased in the xenograft tumor tissues compared with the control group. The Ki67 indexes were 83.2%, 7.4% respectively in control and 50 mg/kg group (Fig. 8F–J). Thus, crowberry inhibits CCA proliferation via *DEK *in vivo.

## Discussion

CCA is a malignant tumor of the digestive tract with a high mortality rate [24, 25]. Due to immunomodulatory, antibacterial, and even antitumor effects, natural plant caused a widespread concern [26]. As previously mentioned, crowberry shows significant potential efficacy on the treatment of various diseases, but its antitumor effects on CCA have not been fully elucidated. In this study, we found that crowberry extracts significantly suppressed the cell proliferation and metastasis of HuCCT1 and QBC939 cells. Particularly, it showed that the 50 mg/kg group tumors exhibited a significant reduction in volume and weight in vivo. The HPLC–MS/MS analysis of crowberry showed obvious peaks for 2'-methoxy-4'-hydroxy-dihydrochalcone (**1**), 2',4'-dihydroxy-dihydrochalcone (**2**), and 2',4'-dihydroxy-chalcone (**3**) contents compared with other plant *Ixeridium gracile* it is higher [27]. Previous studies indicated that 2',4'-dihydroxychalcone suppresses PC-3 human prostate cancer cell growth by inducing apoptosis [28].

Apoptosis induction is one of the key mechanisms of anticancer medicine [29]. Many studies have shown that prohibiting the expression and/or function of Bax can resist cytochrome C (Cyt C) released by mitochondria, prohitbit the reducing mitochondrial membrane potential, and guarantee cells against apoptosis [30]. Consistent with these results, we found that crowberry reduced the expression of the pro-survival protein Bcl-2 but elevated the expression of the pro-apoptotic protein Bax and key mitochondrial apoptotic pathway factor Cyto C. A previous study showed that Cyto C indirectly triggered initiator caspase-9 that is primarily responsible for the beginning of the apoptotic pathway and executioner caspase-3 that is responsible for the definite cleavage of cellular components leading to apoptotic cell death [31]. In general, PARP is considered an indicator of caspase 3 activation. In our study, the protein expressions of cleaved-caspase 9, cleaved-caspase 3, and cleaved-PARP were increased in crowberry-treated CCA cells, further promoting the apoptosis of CCA cells. It has been reported that, in HCT116 cells, 6-gingerol induced apoptosis by upregulating the protein expression of caspase3, PARP1, and Bax and downregulating Bcl-2 [32], further validates our point of view. Overall, crowberry induces apoptosis in CCA cells.

*DEK* is an oncogene that promotes proliferation, EMT, and metastasis in various cancers [33, 34]. Previous studies have shown that *DEK* is highly expressed in CCA and that inhibiting *DEK* gene expression can delay the development of CCA [35]. We found that *DEK* is highly expressed in CCA by using the UALCAN database. In the present study, we found that *DEK* protein levels in CCA cell lines (HuCCT1, QBC939, and RBE) were significantly higher than in the normal human biliary cell line HIBEpic. These results are consistent with UCLAN database analysis. In our study, crowberry significantly inhibited the expression of *DEK* in vivo and vitro. To survey the function of *DEK* in CCA tumorigenesis, we silenced *DEK* expression. Consequences demonstrated that in vitro CCA cell proliferation and migration were inhibited, and as we expected, cell proliferation and migration were enhanced when *DEK* was overexpressed, suggesting an oncogenic role for *DEK* in CCA. To verify that cowberry-modulated cell proliferation is related to *DEK*-induced apoptosis, we considered our previous research results, which showed no change in the CCA cell apoptosis after *DEK* was silenced. We speculated that cowberry-modulated cell death is not related to apoptosis induction, but may be related to Akt signaling pathway inactivation (si*DEK* data are not shown). Yang et al. had previously discovered that *DEK* overpresstion exerted a significant promoting effect on breast cancer cell proliferation, invasion, and migration, and that high *DEK* expression was positively correlated with lymph node metastasis and the Ki67 index, which indicated poor prognosis [12]. Further, the xenograft model confirmed that crowberry treatment significantly reduced the volume of transplanted tumor. IHC staining of tumor tissue showed that Ki67 and *DEK* were reduced. These effects point that *DEK* is a key mediator of the crowberry-induced inhibition of CCA cell proliferation and metastasis in vitro and in vivo, and can be used as a target gene for CCA treatment.

EMT is a progressive biological phenomenon in which epithelial cells gradually obtain a mesenchymal cell phenotype, resulting in enhanced invasion and metastasis [36, 37]. Wang et al. reported that COX-2 induces the loss of E-cadherin expression, resulting in the promotion of ovarian cancer cell invasiveness [38]. Due to tumor migration is associated with EMT process, we analyzed the expression of EMT-associated factors. After being treated with crowberry, our results demonstrated that, in CCA cells, the lateral and vertical migration were obviously decreased. In addition, epithelial marker E-cadherin was upregulated, after treatment with crowberry. In contrast, mesenchymal markers such as Vimentin and Snail were downregulated. Xu et al. found that silencing *DEK* inhibited cervical cancer tumorigenesis and metastasis by downregulating the Wnt/β-catenin pathway [39]. Yang et al. showed that after silencing the *DEK* gene in lung cancer cells, the expression of E-cadherin was upregulated, and the levels of Vimentin and Snail were downregulated [40]. It was also found that *DEK*-dependent migration and EMT occurs via β-catenin/E-cadherin signaling in HCC cells [34]. In accordance with these results, we discovered that the silencing of *DEK* prohibited EMT by reducing the expression of Vimentin and Snail, in contrast, promoting the expression of E-cadherin. When *DEK* was overexpressed, it can accelerate the EMT process.

The Akt/mTOR signaling pathway is an indispensable way that adjusts cell proliferative potential, migration, and invasion [41, 42]. Scholars found that Akt/mTOR signaling pathway is activated in CCA, and the inhibition of this pathway can delay CCA progression [43, 44]. Thus, we investigated the effect of crowberry on the Akt/mTOR signaling pathway. Specifically, in CCA cells the expression of p-Akt, p-S6, and p-4EBP1 was significantly downregulated. Report indicates that *DEK* also stimulates Akt phosphorylation in myeloid-enriched progenitor cells [45]. The GeneMANIA database showed that there is an interaction between *DEK* and Akt/mTOR signaling pathways. Therefore, we urgently sought to determine whether *DEK* regulates the Akt/mTOR signaling pathway to inhibit CCA. Cui suggested that si*DEK* suppresses microglia-mediated neuronal cell death via the downregulation of the Akt pathway [46]. In this study, silencing the *DEK* gene significantly inhibited the expression of p-Akt, p-4EBP1, and p-S6, which is consistent with previous reports [47]_,_ while the outcome of *DEK* overexpression was opposite. Our previous results showed that the *DEK* protein expression level was not changed when an Akt inhibitor was incubated with CCA cells, suggesting that *DEK* is located upstream of the Akt signaling pathway [48]. The researchers showed that p53 transcriptional activity was significantly increased due to *DEK* knockdown. Furthermore, *DEK* can affect different types of p53 target genes at different stages [51]. Kim indicated that p53 simultaneously controls multiple pathways to induce cellular senescence through p21 and Akt [52]. The above indirectly proves that there is an interaction between *DEK* and AKT. However, there are some limitations to this study, the specific mechanism of whether *DEK* and Akt signaling have a direct or indirect action remained to explore in further studies. *DEK* promotes cancer progression through many pathways. Yang indicated that *DEK* promote the proliferation and invasion of breast cancer cells by activating the Wnt signaling pathway [12]. As mentioned above, the proliferation of CCA cells was inhibited after si*DEK* transfection. We speculated that the inhibition of CCA cell proliferation induced by *DEK* is achieved, at least in part, through the Akt signaling pathway. Whether other pathways are involved remains to be further studied. Phosphorylated and activated Akt can regulate the EMT by modulating the actin cytoskeleton in a cell [49]. Song suggested that inhibition of *DEK* notably mitigated the bronchial EMT process in vitro and in vivo, and these effects may be interceded through the PI3K signaling pathway [50]_._ As we mentioned above, si*DEK* can inhibit the migration, EMT process and Akt signaling pathway in CCA cells, therefore, we have reason to believe that activation of the PI3K/AKT/mTOR signaling pathway is required for *DEK*-stimulated CCA cell proliferation and migration.

## Conclusion

In summary, we demonstrated that crowberry inhibited CCA cell proliferation and migration through a molecular mechanism that includes inhibition of *DEK* and the Akt signaling pathway in vivo and vitro. Importantly, these data provide novel insights into the mechanisms of CCA and indicate that crowberry may be a novel agent for the development of therapies to improve CCA clinical treatment and prognosis. However, identifying the specific component of crowberry that is responsible for its antitumor effect still requires further research.

## Supplementary Information

**Additional file 1:** Crowberry suppresses the migration and EMT of CCA cells.

## Data Availability

The datasets used and/or analyzed during the current study are available from the corresponding author upon reasonable request.

## Abbreviations

Akt: Serine/threonine Kinase
CCA: Cholangiocarcinoma
Cyt C: Cytochrome C
Caspase: Cysteinyl aspartate specific proteinase
EMT: Epithelial-to-mesenchymal transition
EdU: 5-Ethynyl-2'-deoxyuridine
HPLC: High-performance liquid chromatography
ig: Intragastric administration
MTT: 3-(4,5)-Dimethylthiahiazo (-z-y1)-3,5-di- phenytetrazoliumromide
PARP: Poly (ADP-ribose) polymerase
siRNA: Small interfering RNA
