# Supplementary material for: Crowberry inhibits cell proliferation and migration through a molecular mechanism that includes inhibition of DEK and Akt signaling in cholangiocarcinoma
Source: Chin Med. 2022 Jun 13;17:69. doi: 10.1186/s13020-022-00623-6 (PMC9190153; doi:10.1186/s13020-022-00623-6)
Supplement: Supplementary file 1 — Additional file 1: Crowberry suppresses the migration and EMT of CCA cells. [file 13020_2022_623_MOESM1_ESM.pdf]

Supplementary Fig.

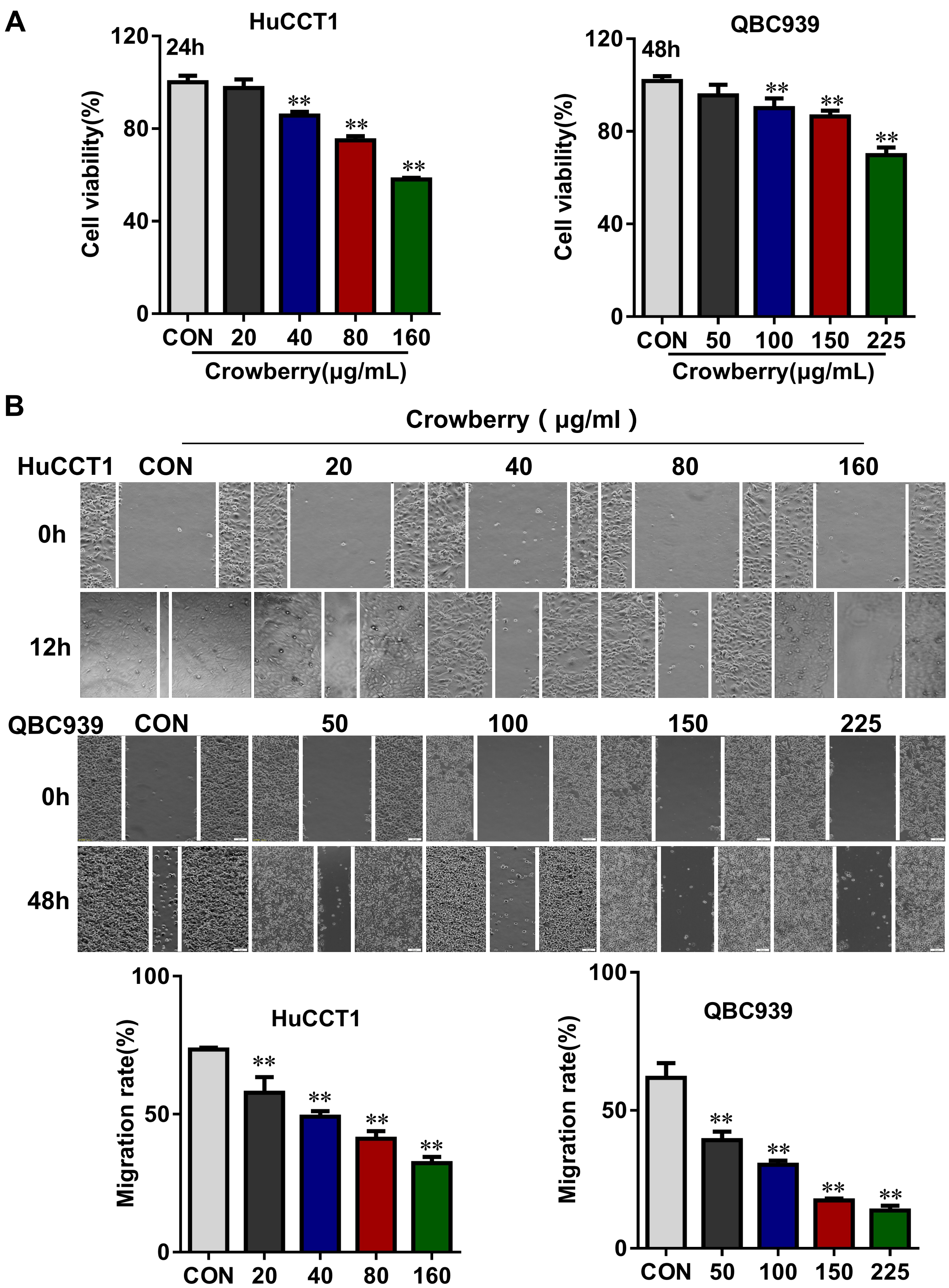

Figure Captions

MTT assay was used to detected the effect of crowberry on the viability in HuCCT1 and QBC939 cells (A) **P < 0.01 (vs CON group, n=3). Horizontal migration ability of HuCCT1 and QBC939 cells (B)P < 0.01 (vs CON group, n=3), ×200. CON, control.**
